# Acute Exacerbation of Hypereosinophilic Syndrome Complicated With Dermatitis, Enteritis, and Myositis: A Case Report

**DOI:** 10.7759/cureus.34090

**Published:** 2023-01-23

**Authors:** Yuki Takebuchi, Airi Minatogawa, Yumi Naito, Chiaki Sano, Ryuichi Ohta

**Affiliations:** 1 Family Medicine, Shimane University Medical School, Izumo, JPN; 2 Family Medicine, International University of Health and Welfare, Tokyo, JPN; 3 Community Care, Unnan City Hospital, Unnan, JPN; 4 Community Medicine Management, Faculty of Medicine, Shimane University Medical School, Izumo, JPN

**Keywords:** myalgia, acute onset weakness, eosinophilic myositis with chronic diarrhea, chronic urticaria, eosinophilic gastroenteropathy, abnormal rise of eosinophils, hypereosinophilic syndrome, rural hospital, general medicine

## Abstract

Hypereosinophilic syndrome is a disease that presents with a variety of symptoms caused by an abnormal rise in eosinophils in the blood and infiltration into various organs. Typical symptoms include skin symptoms and diarrhea. Diagnosis may be difficult because of the self-limiting phases of the disease with various symptoms. In addition, diagnosis may be delayed by the presence of rare concomitant symptoms, such as muscle pain and numbness. Here, we report the case of a 67-year-old patient with asymptomatic hypereosinophilia with chronic diarrhea, acute-onset weakness, and myalgia. We diagnosed eosinophilic gastroenteropathy, chronic urticaria, and eosinophilic myositis through multiple biopsies of the skin and colon. This case shows that chronic hypereosinophilic syndrome can be exacerbated transiently with acute symptoms and that prompt examination and treatment of hypereosinophilic syndrome based on the involved organs is recommended in a background of eosinophilia.

## Introduction

Hypereosinophilic syndrome is a disease in which eosinophils increase despite the absence of eosinophilic factors and induce multiple organ dysfunction syndromes due to eosinophil infiltration. Eosinophilia is defined as an eosinophil count ≥ 1500 cells/μL in peripheral blood at two examinations at least one month apart or pathological confirmation by tissue staining. Eosinophilia can be classified into four categories: hereditary with a family history, primary (clonal/neoplastic) with the clonal proliferation of eosinophils, secondary to a cytokine-mediated reactive increase of non-clonal eosinophils because of underlying disease, and idiopathic with none of the above [1]. However, the extent to which eosinophils contribute to clinical conditions and complications has not been elucidated. Hypereosinophilic syndrome is a very rare disease, with an estimated prevalence of 0.36-6.3 per 100,000 persons [2]. Many patients are diagnosed at 20-50 years old, and the syndrome can also occur in children [3-5].

In hypereosinophilic syndrome, eosinophils infiltrate and destroy various tissues. The general target organs are the skin, lungs, and gastrointestinal tract. Eosinophil infiltration can also cause cardiovascular and brain damage in rare cases. Gastrointestinal disorders caused by eosinophilia can lead to chronic diarrhea, impaired potassium absorption, and hypokalemia [6]. In rare cases, eosinophils can cause eosinophilic fasciitis and dermatitis, which can be difficult to diagnose accurately because they follow various clinical courses and require high doses of steroids [7,8]. In this report, we discuss the case of a patient with an extensive rash, chronic diarrhea, lymphadenopathy, hypokalemia, and periodic paralysis, who was finally diagnosed with hypereosinophilic syndrome that involved the skin, intestine, and muscles. The patient was treated with an antihistamine and did not require systemic steroid therapy. This case report shows the difficulties in diagnosing an exacerbation of chronic hypereosinophilic syndrome with multiple symptoms and involved organs and the possibility of less intensive treatment.

## Case presentation

A 67-year-old man visited a rural community hospital with a chief complaint of lower back pain and generalized weakness. He had chronic back pain several months before the presentation. The pain gradually worsened one week before he arrived at the hospital. He also had intermittent watery diarrhea for one month. The day before his visit, he developed left lower limb weakness. On the day of his visit, he became aware of the whole-body weakness, which made it difficult for him to move. He called an ambulance to be transferred to the hospital's emergency department (ED) and was admitted to the Department of General Medicine. His medical history included chronic alcohol consumption. He had not taken any medication or over-the-counter drugs for one month.

On arrival at the ED, vital signs were as follows: temperature, 36.5°C; heart rate, 73 bpm; blood pressure, 129/74 mmHg; respiratory rate, 25 breaths/min; SpO2, 99% on room air; and he was alert and fully oriented. Skin examinations revealed extensive eczema. Manual muscle strength testing revealed deltoid 5/5, biceps 5/5 (right/left), triceps 4/3 (right/left), flexor carpi radialis 4/4 (right/left), extensor carpi radialis 5/5 (right/left), iliopsoas 3/2 (right/left), quadriceps 4/2 (right/left), flexor carpi radialis 4/2 (right/left), tibialis anterior 5/5 (right/left), and triceps femoris 5/5 (right/left). Tendon hyperreflexia was not observed in the upper or lower extremities, and the straight leg raising test was 80/45° (right/left). The same test revealed pain in the medial aspect of the left thigh. The initial blood findings showed hypokalemia, the elevation of liver enzymes, eosinophil, and elevated creatinine kinase. In addition, the blood gas test showed metabolic alkalosis (Table 1).

**Table 1:** Initial laboratory data of the patient

Parameter	Level	Reference
White blood cells	8.50	3.5-9.8 × 10^3^/μL
Neutrophils	58.5	44.0-72.0%
Lymphocytes	15.2	18.0-59.0%
Monocytes	9.1	0.0-12.0%
Eosinophil	16.8	0.0-10.0%
Basophils	0.4	0.0–3.0%
Red blood cells	3.82	4.10-5.30 × 10^6^/μL
Hemoglobin	13.0	13.5-17.6 g/dL
Hematocrit	37.1	36-48%
Mean corpuscular volume	97.2	82-101 fl
Platelets	26.5	13.0-36.9 × 10^4^/μL
Total protein	5.8	6.6-8.1 g/dL
Albumin	3.0	3.9-4.9 g/dL
Total bilirubin	1.2	0.2-1.2 mg/dL
Aspartate aminotransferase	117	8-38 IU/L
Alanine aminotransferase	55	4-44 IU/L
γ-Glutamyl transpeptidase	80	16-73 IU/L
Lactate dehydrogenase	478	106-211 U/L
Blood urea nitrogen	4.8	8.0-20.0 mg/dL
Creatinine	0.70	0.40-1.10 mg/dL
Estimated glomerular filtration rate	89.8	>60.0 mL/min/L
Serum sodium	143	135-147 mEq/L
Serum potassium	1.7	3.3-4.8 mEq/L
Serum Chloride	99	98-108 mEq/L
Serum Calcium	8.5	8.8-10.2 mEq/L
Serum Phosphorus	1.5	2.7-4.6 mEq/L
Serum Magnesium	1.8	1.8-2.3 mEq/L
Creatinine kinase	4153	56-244 U/L
C-reactive protein	1.51	<0.30 mg/dL
Thyroid-stimulating hormone	1.91	0.35–4.94 μIU/mL
Free T3	2.1	1.88-3.18 ng/dL
Free T4	1.0	0.70–1.48 ng/dL
Immunoglobin G	1024	870–1700 mg/dL
Immunoglobin M	74	35–220 mg/dL
Immunoglobin A	221	110–410 mg/dL
pH	7.455	7.35-7.45
pCO_2_	47.9	35-45 mmHg
HCO_3_	32.6	20-28 mmol/L

Electrocardiography (EKG) showed QT prolongation, which was caused by hypokalemia. The stool test for oocysts investigating parasitic infection was negative. A central venous catheter was inserted to correct this, and an intravenous potassium infusion of 100 mEq was started. On the day after admission, intravenous potassium infusion was increased to 200 mEq owing to low serum potassium of 1.9 mEq/L. His stools were soft. On the second day of admission, intravenous potassium infusion was reduced to 120 mEq/L because of serum potassium of 2.9 mEq/L and persistent soft stools. On the third day of admission, intravenous potassium infusion was reduced to 60 mEq, and the stools were soft. On the fourth day of admission, serum potassium level returned to 4.0 mEq/L with the disappearance of diarrhea and the normalization of EKG. On the seventh day of admission, serum potassium level became 4.5 mEq/L. In contrast, weakness in the arms and legs improved with the normalization of serum potassium levels. Metabolic alkalosis also improved with an improvement in diarrhea.

**Figure 1:**
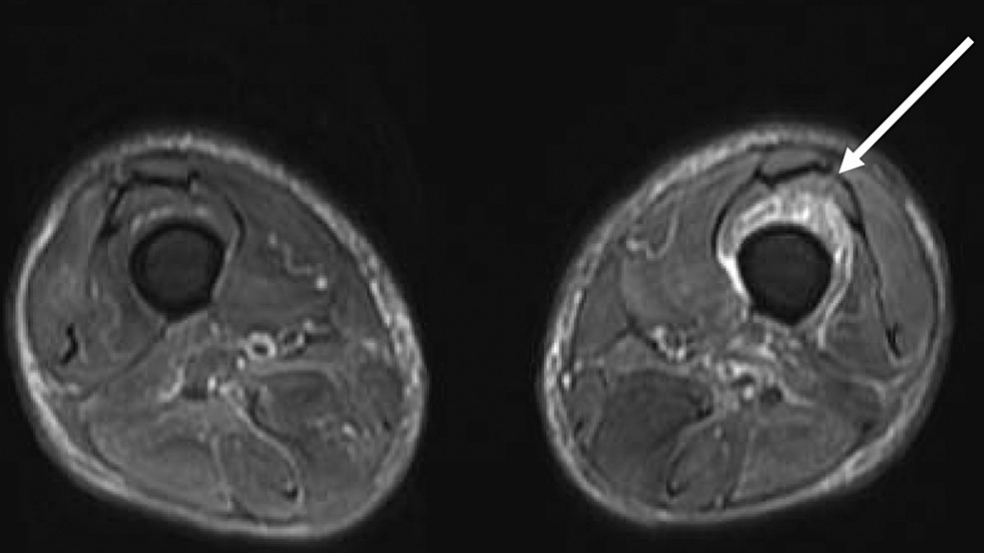
Magnetic resonance imaging (Short-TI Inversion Recovery) showing high signal in the vastus medialis muscle of the left (white arrow)

On the day of admission, his white blood cell count was 8500/μL with 16.8% (1428) eosinophils. We suspected eosinophilic enterocolitis and fasciitis due to extensive eczema, chronic diarrhea, weakness, and pain in the left thigh. Contrast-enhanced computed tomography of the chest and pelvis was performed to investigate lymphoma and other inflammatory conditions in the deep parts, and mediastinal and hilar areas and bilateral inguinal lymphadenopathy were observed. Magnetic resonance imaging showed a high signal intensity in the vastus intermedius muscle (Figure 1).

**Figure 2:**
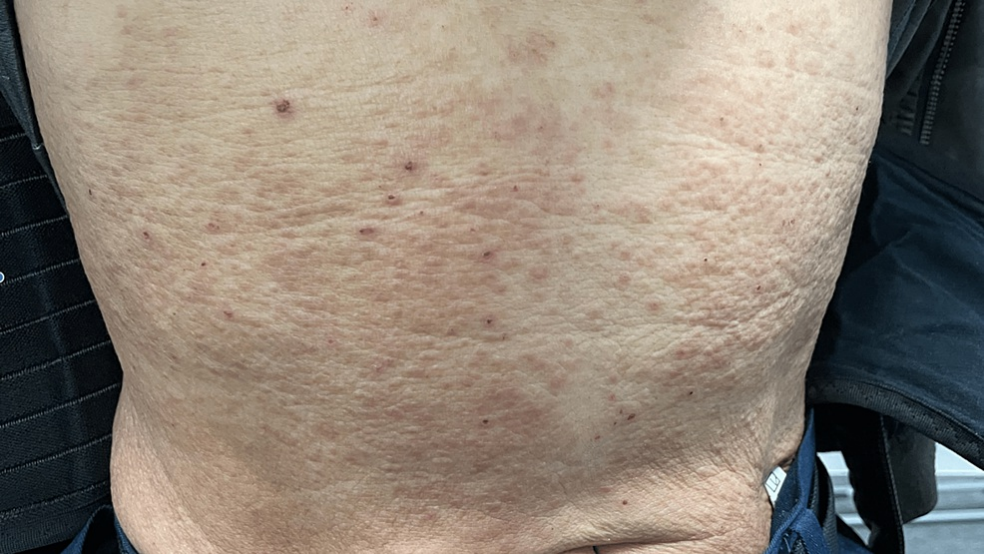
Skin rash observed on the patient’s abdomen.

Chronic dermatitis predominating the trunk and extremities was observed (Figure 2), and a biopsy was performed to differentiate between atopic dermatitis and eosinophilic cellulitis.

A skin biopsy showed infiltration of eosinophils into the subcutaneous tissue. A total colonoscopy was performed to investigate chronic diarrhea, and a mucosal biopsy was performed. Furthermore, an inguinal lymph node biopsy was performed. We confirmed the presence of eosinophil infiltration and the absence of obvious malignant findings. We diagnosed the patient with hypereosinophilic syndrome with multiple organ damage. Owing to the lack of symptom exacerbation, we treated the patient with antihistamines and symptomatic therapy without steroids. The patient's symptoms gradually improved, and he was discharged after 14 days of hospitalization.

## Discussion

In the present case, the patient was diagnosed with hypereosinophilic syndrome due to symptoms of diarrhea, chronic eczema, and weakness. Diarrhea can be caused by gastric mucosal damage caused by eosinophilia. Chronic diarrhea may have caused a loss of acid from the body. Diarrhea-induced metabolic alkalosis with hypokalemia may cause weakness. Lower leg pain and weakness may also be caused by hypokalemia and infiltration of eosinophils into the muscles. This case suggests that acute symptoms can exacerbate chronic eosinophilia. Therefore, prompt medical examination and treatment of eosinophilia symptoms are necessary.

Chronic eosinophilia is a slowly worsening disease, with clinical manifestations resembling acute exacerbation. Therefore, follow-up for eosinophilia should be performed with caution [6,7]. In our case, the patient experienced a rapid exacerbation of his chronic eosinophilia. Hypereosinophilic syndrome can be fatal when the heart and lungs are the main sites of involvement [8]. In contrast, symptoms that appear slowly on the skin or in the gastrointestinal tract can often be treated symptomatically, and strong treatments such as immunosuppressive drugs are rarely used [3]. Furthermore, when eosinophilia remains uncontrolled, a chronic progressive inflammatory condition may develop systematically, including in the muscles. Our patient's condition was associated with a high degree of diarrhea, which eventually led to severe damage to the intestinal mucosa and persistent diarrheal symptoms [6]. As in our case, this can lead to severe hypokalemia and should be treated cautiously. Even if the early symptoms of eosinophilia are mild, appropriate follow-up and treatment to control eosinophilia are necessary.

Eosinophilia can affect various organs, and the diversity of its clinical course can make diagnosis difficult and demand the differentiation of hematological and parasitic diseases. In the present case, we ruled out leukemia, lymphoma, and parasitic diseases through the blood smear, lymph node biopsy, and oocysts tests of stool. Besides, the patient initially had only mild skin and gastrointestinal symptoms. Still, as the disease progressed, the symptoms worsened in various organs and infiltrated new organs, such as the muscles [6,7]. Eosinophilia is often a slow disease that resolves spontaneously. In contrast, persistent eosinophilia is a progressive condition. If not treated with immunosuppression or other therapies, the disease may continue progressing, leading to multiorgan damage, as in our case [8]. In the early stages, symptoms in various organs may be missed because of the slowly progressive nature of the disease. 

Early detection of critical diseases depends on a patient's effective help-seeking behavior. As in this case, general physicians need to approach complicated symptoms during the first contact as a system-specific specialist [9]. They have to take a systemic approach to diagnosis when faced with complicated symptoms at the initial visit, considering the usage of immunosuppressants. In rural hospitals, general physicians deal with multimorbidity among older and more complicated patients [10]. General physicians should deal with patients with multiple symptoms comprehensively [11]. However, rural communities in Japan currently do not have enough general physicians to support their needs [12,13]. Therefore, it is important to improve patients’ help-seeking behaviors for prompt diagnosis of critical diseases and improve the education of general physicians in Japan.

## Conclusions

Chronic eosinophilia is a progressive condition that sometimes develops into acute exacerbations. Early diagnosis and treatment of the underlying cause of eosinophilia, which is often an indeterminate complaint, can slow disease progression. In the future, it will be necessary to improve the diagnostic ability of general practitioners in the community. Appropriate patient behavior should be encouraged to promote early diagnosis and treatment.
